# Reversible Cerebral Vasoconstriction Syndrome Associated With Vortioxetine: A Case Report

**DOI:** 10.7759/cureus.114868

**Published:** 2026-08-20

**Authors:** Beatriz Fonseca Silva, Pedro Felgueiras, André Oliveira, Lúcia Ribeiro

**Affiliations:** 1 Psychiatry, Unidade Local de Saúde Gaia/Espinho, Vila Nova de Gaia, PRT; 2 Psychiatry, Unidade Local de Saúde de Matosinhos, Matosinhos, PRT; 3 Liaison Psychiatry Unit, Vila Nova de Gaia/Espinho Hospital Center, Vila Nova de Gaia, PRT; 4 Liaison Psychiatry Unit, Unidade Local de Saúde Gaia/Espinho, Vila Nova de Gaia, PRT

**Keywords:** consultation liaison psychiatry, management of depression in chronic illness, reversible cerebral vasocontriction syndrome, serotonin modulator, serotonin reuptake inhibitors

## Abstract

Reversible cerebrovascular syndrome (RCVS) is a condition that is clinically characterized by sudden and severe headaches, and its imaging hallmark consists of multifocal phenomena of reversible cerebral vasoconstriction. Multiple triggers are associated with its occurrence, including many types of medication.

A 55-year-old woman with a history of recurrent depressive disorder treated with vortioxetine presented with a sudden-onset thunderclap headache triggered by physical exertion. Neurological examination was unremarkable. CT angiography demonstrated multifocal segmental irregularities involving the bilateral middle cerebral artery branches and pericallosal vessels, consistent with RCVS. No alternative precipitating factors were identified. Vortioxetine was discontinued and replaced with trazodone following Liaison Psychiatry consultation. The patient experienced complete clinical recovery with supportive treatment and was discharged without neurological deficits.

This case highlights vortioxetine as a possible precipitating factor for RCVS and supports the growing evidence implicating serotonergic antidepressants in the syndrome. Clinicians should consider RCVS in patients presenting with a thunderclap headache, even after a single episode and in the absence of focal neurological deficits. Prompt recognition, vascular imaging, and withdrawal of the suspected precipitating agent are essential to minimize the risk of neurological complications. Further case reports and pharmacovigilance studies are needed to better define the relationship between vortioxetine and RCVS.

## Introduction

Reversible cerebral vasoconstriction syndrome (RCVS) is a rare and frequently underrecognized condition that comprises a cluster of disorders characterized by sudden, severe headaches associated with diffuse and predominantly reversible cerebral arterial vasoconstriction [1, 2]. These headaches typically reach peak intensity within the first minute, often presenting as “thunderclap” headaches. They recur in up to 85%-90% of patients, with a gradual reduction in both intensity and frequency over a period of four weeks [3, 4]. Within the first 10 days of recurrent episodes, patients may develop seizures or focal neurological deficits [1, 4]. Visual disturbances are also common and may include blurred vision, cortical blindness, and scotomas [3].

RCVS is classically defined by multifocal, segmental vasospasms of the cerebral arteries [5]. However, the relationship between headache occurrence and the dynamic change in arterial caliber remains poorly understood. The severity and timing of headache episodes do not consistently correlate with the degree of angiographic vasoconstriction, suggesting that mechanisms beyond mere arterial narrowing contribute to symptom emergence [6]. Proposed mechanisms include dysfunction of trigeminovascular and serotoninergic pathways linking cerebral vessels to the brainstem [3, 6]. These pathways are involved in the regulation of cerebrovascular and nociceptive signaling, and their dysregulation may result in both abnormal vasomotor responses and exaggerated pain perception [6]. Additionally, endothelial dysfunction (predisposing cerebral arteries to transient vasospasm), sympathetic overactivity (associated with heightened vascular smooth muscle contraction), and oxidative stress are considered key contributors to its pathophysiology [6, 7]. Although typically benign and self-limited, RCVS can lead to severe complications such as ischemic stroke, subarachnoid hemorrhage, and posterior reversible encephalopathy syndrome. These primarily occur within the first week after headache onset [3].

There is a well-established association between RCVS and multiple potential triggers, with certain medications and vasoactive substances playing a particularly prominent role [3]. Indeed, more than half of RCVS cases occur after exposure to drugs with adrenergic or serotonergic activity [8, 9]. These agents interfere with physiological regulation of cerebral vascular tone by enhancing sympathetic activity or altering serotonergic signaling, thereby promoting excessive and transient arterial vasoconstriction in susceptible individuals [10]. The association with serotonergic and adrenergic substances further supports the hypothesis that an imbalance in cerebrovascular tone and autonomic regulation represents a central component of RCVS pathophysiology [9]. 

The actual incidence and prevalence of RCVS are not known, but it appears to be increasing, probably due to a combination of increased awareness among clinicians and the widespread use of MRI and vascular imaging. It predominantly affects middle-aged women, with female-to-male ratios ranging from 2:1 to 10:1 [3]. Diagnosis is based on clinical presentation, exclusion of other potential causes, and documentation of multifocal vasoconstriction of the cerebral arteries by angiography and of its reversibility within 12 weeks of onset [11]. The angiographic appearance of affected arteries in RCVS is classically a combination of smoothly tapered narrowing alternating with dilatation, producing the characteristic "sausage on a string" appearance. Lesions tend to progress centripetally and are primarily seen in medium to large arteries [3, 4].

Vortioxetine is a multimodal antidepressant classified as a serotonin modulator and stimulator. It exerts its pharmacological effects through simultaneous actions at six serotonergic targets via three distinct mechanisms: inhibition of the serotonin transporter, antagonism of the 5-HT3 receptor, and modulation of several G protein-coupled serotonin receptors, acting as a 5-HT1A agonist, 5-HT1B partial agonist, and 5-HT1D and 5-HT7 antagonist [5].

Given the established association between RCVS and exposure to serotonergic agents, we report the case of a female patient who presented to the emergency department with a single thunderclap headache and was subsequently diagnosed with RCVS, likely associated with vortioxetine use. 

## Case presentation

We present the case of a 55-year-old female patient who presented to the emergency department with sudden-onset holocranial headache that reached maximum intensity (9.5 out of 10) in a few seconds after exercising. Additionally, mild nausea and photophobia were reported. There were no signs of hemodynamic instability or fever, and the neurological exam was unremarkable. She had a medical history of type 2 diabetes, migraine, dyslipidemia, post-menopausal status, and recurrent depressive disorder (with regular follow-up by psychiatry). Concerning the migraine diagnosis, the patient was episode-free for over twenty years and had not been taking any migraine prophylaxis. There was no documented personal or family history of neurocognitive disorders or cardiovascular events. There was no history of alcohol, tobacco, or other substance use. She was recently medicated with vortioxetine 10 mg, after multiple trials with other antidepressants. 

**Figure 1:**
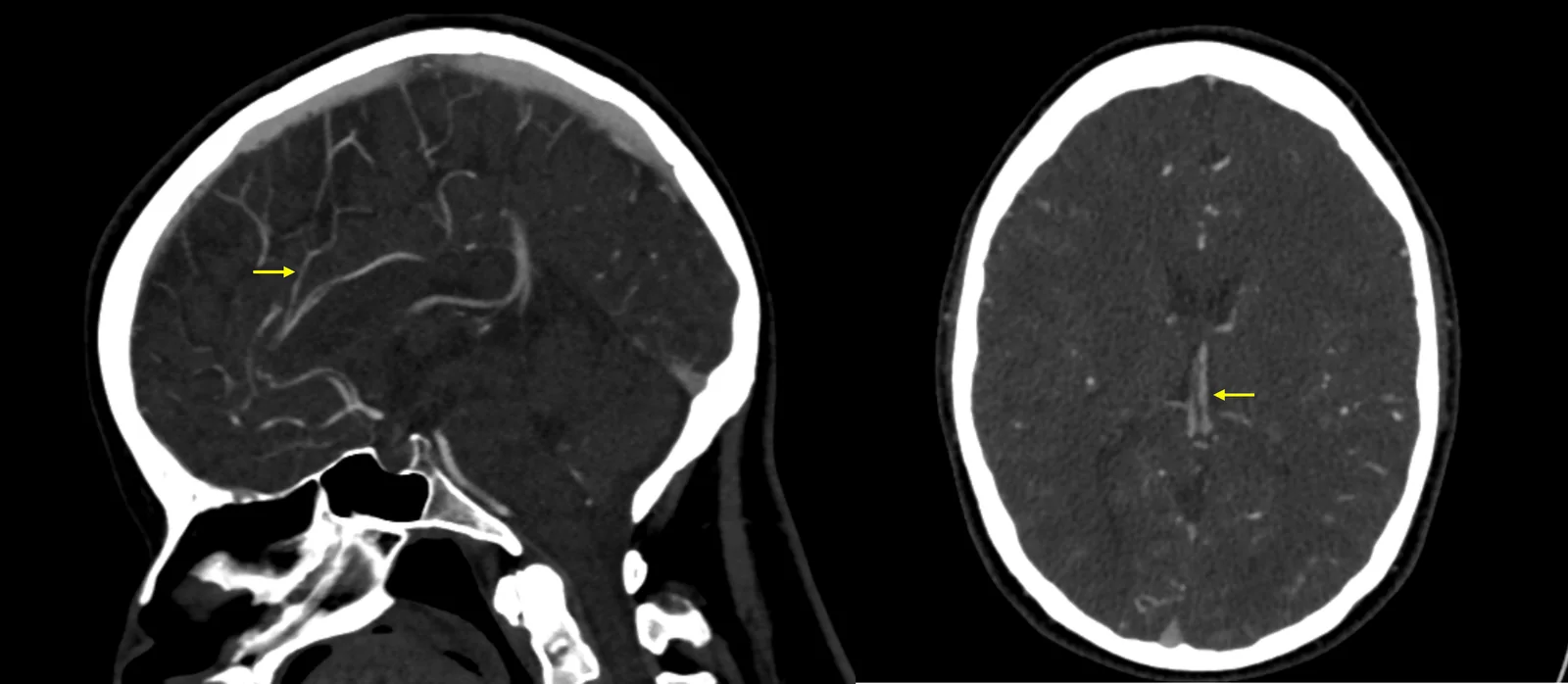
Head CT images showing obliterative irregularities on cortical arteries and the pericallosal arteries (sagittal and axial planes).

A head CT scan and angiography were performed (Figure 1). Non-obliterative irregularities in the M2 branches of both middle cerebral arteries and in the pericallosal and callosomarginal arteries and a diffuse prominence of bilateral parieto-occipital cortical arteries were documented. There was an absence of significant intracranial or cervical atheromatosis. 

A neurology consult was requested, and, due to the suspicion of RCVS, the patient was admitted to the inpatient department for additional diagnostic work and clinical surveillance. Further blood tests (Table 1) and cervical and transcranial Doppler ultrasounds were performed without relevant findings. 

**Table 1:** Laboratory analysis results of the patient

Parameter	Value	Reference values
Hemoglobin	13.4 g/dL	12.0-16.0 g/dL
Erythrocytes	4.34 x 10E6/uL	3.8-5.2 x 10E6/uL
Leukocytes	8,89 x 10E3/uL	3.6-11.0 x 10E3/uL
Lymphocytes	4.00 x 10E3/uL	1.0-4.8 10E3/uL
Monocytes	7.0 x 10E3/uL	0.1-0.8 x 10E3/uL
Platelets	290 x 10E3/uL	150-440 x 10E3/uL
Glucose	74 mg/dL	60-100 mg/dL
Creatinine	0.71 mg/dL	0.51-0.95 mg/dL
Urea	30 mg/dL	13-43 mg/dL
Sodium	141 mmol/L	136-145 mmol/L
Potassium	3.57 mmol/L	3.5-5.0 mmol/L
Chloride	102.0 mmol/L	98-107 mmol/L
Total calcium	9.3 mg/dL	8.4-9.7 mg/dL
Uric acid	5.2 mg/dL	0.2-5.7 mg/dL
Total bilirubin	0,29 mg/dL	0.1-1.1 mg/dL
Lactate dehydrogenase	143 U/L	135-214 U/L
Aspartate aminotransferase	22 U/L	4-27 U/L
Alanine aminotransferase	19 U/L	4-34 U/L
Gamma-glutamyltransferase	37 U/L	5-61 U/L
Albumin	4.42 g/dl	4.2-5.1 g/dL
Total proteins	7.1 g/dL	6.4-8.3 g/dL
Total cholesterol	152 mg/dL	120-200 mg/dL
Triglycerides	79 mg/dL	<200 mg/dL
High-density lipoprotein cholesterol	48 mg/dL	55-120 mg/dL
Low-density lipoprotein cholesterol	88 mg/dL	3-100 mg/dL
Thyroid-stimulating hormone	3.46 uUI/mL	0.27-4.2 uUI/mL
Free thyroxine	1.39 ng/dL	0.93-1.70 ng/dL
C-reactive protein	0.36 mg/dL	0-0.5 mg/dL
Erythrocyte sedimentation rate	11 mm/Hr	0-20 mm/Hr
HbA1c	38 mmol/mol	20-42 mmol/mol
Creatine kinase-myocardial band	2.8 ng/mL	0.1-3.77 ng/mL
Troponin T	5 ng/L	5-14 ng/L
Fibrinogen	321 mg/dl	200-400 mg/dL
Activated partial thromboplastin time	29.5 seconds	25.2-35.2 seconds
D-dimer	0.22 ug/ml	<0.5 ug/mL
Prothrombin time	12.4 seconds	11.5-14.5 seconds
Hepatitis B antigens and antibodies	Negative	
HIV-1 and 2 antigens and antibodies	Negative	
Anti-treponema antibody (electrochemiluminescence immunoassay)	Nonreactive	
Hepatitis C antibody	Negative	
Urine summary analysis	No relevant alterations	

The only identifiable possible precipitant for the clinical picture, besides the exercise, was the use of daily vortioxetine by the patient. Because of this, a Liaison Psychiatry consult was requested for the pharmacological management of the patient’s depressive syndrome. Considering the suspected association between serotoninergic components and RCVS, antidepressant switching to other options (in this case, trazodone) was suggested. Antidepressant withdrawal was not considered an alternative due to the patient maintaining mild depressive symptomatology and the increased risk of worsening in the near future if left untreated. 

During the hospitalization period, the patient gradually improved under analgesic therapy until the headache had resolved. She always remained hemodynamically stable and without any neurological deficits or complications. A magnetic resonance angiography was conducted eight days after admission, which revealed no evidence of vasoconstriction or any change in vessel caliber, confirming the resolution of the event. 

The patient was discharged with neurology and psychiatry follow-up scheduled and the recommendation to avoid high-intensity physical exercise until further notice.

## Discussion

The present case illustrates the cardinal features of RCVS, as the patient presented with a thunderclap headache, underwent an angiography that demonstrated multifocal segmental cerebral artery vasoconstriction, and had other potential systemic causes excluded. Indeed, concordant with the available literature, headache is the most common presenting symptom of this syndrome, and it can be the only clinical manifestation [11]. Focal neurological deficits are less frequent [7], and often the neurological examination allows the exclusion of other diagnoses [3]. Diagnosing RCVS can present as a challenge given the vast number of possible neurological entities associated with cerebral ischemia [1]. The patient’s history of multiple cardiovascular risk factors (dyslipidemia and diabetes) and sudden onset during exercise complicated the diagnostic interpretation. 

The pathophysiology of RCVS remains incompletely understood. Current evidence suggests that transient dysregulation of cerebral vascular tone results in multifocal vasoconstriction, likely mediated by mechanisms including endothelial dysfunction, sympathetic overactivity, and oxidative stress [6, 11]. A precipitating factor is identified in approximately 25%-60% of cases, with many recognized triggers associated with enhanced sympathetic activation [7]. Common precipitants include the postpartum state, eclampsia, and exposure to medications, particularly vasoactive and chemotherapeutic agents. Among pharmacological triggers, serotonergic drugs, including medications frequently prescribed in psychiatric practice, have been increasingly implicated in the development of RCVS [3]. The vasoconstrictive effects of serotonin (5-HT) on the cerebral vasculature are well-documented. Serotonin-induced vasoconstriction is thought to be mediated primarily through activation of 5-HT1B and 5-HT2A receptors expressed on vascular smooth muscle cells [1]. The vasoactive and sympathomimetic properties of selective serotonin reuptake inhibitors (SSRIs) have therefore been proposed as the principal mechanism of SSRI-associated RCVS. Current evidence suggests that the risk is not restricted to a particular SSRI and appears to be independent of the specific agent, dosage, or duration of treatment [7].

The available literature consists predominantly of isolated case reports and small case series. Among SSRIs, RCVS has been described following treatment with fluoxetine [1], escitalopram [7], citalopram [12], and paroxetine [13], supporting the hypothesis of a class effect rather than toxicity attributable to a single compound. In contrast, published evidence for other antidepressants remains limited. To our knowledge, no cases of RCVS have been reported with trazodone monotherapy, with the few available reports involving concomitant use of trazodone and another serotonergic antidepressant, precluding attribution to trazodone alone [9]. Similarly, only a single case involving mirtazapine has been described, occurring in the setting of an overdose, making it difficult to extrapolate the risk to therapeutic use [14]. Cases have also been described with bupropion, which acts through norepinephrine and dopamine reuptake inhibition [9, 15]. Overall, the current literature suggests that although serotonergic antidepressants are recognized precipitants of RCVS, the strength of evidence varies considerably between individual agents and is largely based on anecdotal reports rather than systematic studies.

Unlike conventional SSRIs, vortioxetine exerts multimodal serotonergic activity through both serotonin transporter inhibition and direct modulation of multiple serotonin receptor subtypes. Although its receptor profile differs from that of traditional SSRIs, enhancement of serotonergic neurotransmission may still promote cerebral vasoconstriction through activation of vascular serotonin receptors [16]. Evidence linking vortioxetine to RCVS remains limited; however, a published case report of Call-Fleming syndrome (reversible cerebral vasoconstriction syndrome) occurring in temporal association with vortioxetine treatment has suggested a possible association between the drug and the development of RCVS [5]. Although causality cannot be established from an isolated case, this report supports the biological plausibility that vortioxetine may share the class effect observed with other serotonergic antidepressants. The present case further contributes to this limited body of evidence and highlights the need for increased awareness of this potential adverse drug reaction.

Current treatment approaches to RCVS are predicated on recognizing it as a predominantly benign and self-limited condition [3]. The main aspect of treatment is identification and discontinuation of the potential triggers, emphasizing abstinence from common precipitants [12, 17]. The use of long-term antiepileptic agents is generally unwarranted in the absence of irreversible brain injury, even when structural brain lesions are present. Additionally, the use of laxatives and temporary avoidance of physical exertion seem to be beneficial [3].

While many patients with RCVS recover fully without residual symptoms (as vasoconstriction often resolves spontaneously), angiographic features may not always resolve concurrently [3, 18]. When RCVS is caused by antidepressant use, the recurrence of symptoms is low after medication discontinuation [19]. However, there are no studies on the risk of recurrent RCVS when a precipitating agent like SSRI has been restarted, and one study detailing long-term outcomes after RCVS found that up to 26% of patients experienced moderate to severe depression, which highlights the need for continued antidepressant treatment [20].

Selecting an alternative antidepressant after serotonergic drug-associated RCVS remains challenging, as evidence guiding pharmacological management is scarce. Although non-serotonergic agents may represent reasonable alternatives, their safety in this context has not been systematically evaluated [1]. Consequently, treatment decisions should be individualized through close collaboration between psychiatry and neurology departments, balancing the risk of recurrent cerebrovascular events against the need for effective treatment of depressive symptoms. 

## Conclusions

This case adds to the limited literature suggesting a possible association between vortioxetine and reversible cerebral vasoconstriction syndrome. Although a causal relationship cannot be established from a single case, the temporal association, exclusion of alternative precipitants, and clinical improvement following drug discontinuation support vortioxetine as the most likely trigger.

Given the increasing use of serotonergic antidepressants, clinicians should maintain a high index of suspicion for RCVS in patients presenting with thunderclap headache, even when neurological examination and initial investigations are unremarkable. Early recognition, prompt vascular imaging, and discontinuation of the suspected precipitating agent are essential to reduce the risk of potentially serious neurological complications. Further pharmacovigilance data and additional case reports are needed to better characterize the relationship between vortioxetine and RCVS and to guide the management of depressive disorders in affected patients.
